# Magnetic-Resonance-Imaging Texture Analysis Predicts Early Progression in Rectal Cancer Patients Undergoing Neoadjuvant Chemoradiation

**DOI:** 10.1155/2019/8505798

**Published:** 2019-01-17

**Authors:** Valerio Nardone, Alfonso Reginelli, Fernando Scala, Salvatore Francesco Carbone, Maria Antonietta Mazzei, Lucio Sebaste, Tommaso Carfagno, Giuseppe Battaglia, Pierpaolo Pastina, Pierpaolo Correale, Paolo Tini, Gianluca Pellino, Salvatore Cappabianca, Luigi Pirtoli

**Affiliations:** ^1^Istituto Toscano Tumori, Florence, Italy; ^2^Unit of Radiation Oncology, University Hospital of Siena, Siena, Italy; ^3^Department of Precision Medicine, University of Campania “Luigi Vanvitelli”, Naples, Italy; ^4^Unit of Medical Imaging, University Hospital of Siena, Siena, Italy; ^5^Unit of Medical Oncology, Grand Metropolitan Hospital “Bianchi Melacrino Morelli”, Reggio Calabria, Italy; ^6^Sbarro Health Research Organization, Temple University, Philadelphia, PA, USA; ^7^Unit of Colon-Rectal Surgery, University of Campania “Luigi Vanvitelli”, Naples, Italy; ^8^Department of Biology, College of Science and Technology, Temple University, Philadelphia, PA, USA

## Abstract

**Background:**

We hypothesized that texture analysis (TA) from the preoperative MRI can predict early disease progression (ePD), defined as the percentage of patients who relapsed or showed distant metastasis within three months from the radical surgery, in patients with locally advanced rectal cancer (LARC, stage II and III, AJCC) undergoing neoadjuvant chemoradiotherapy (C-RT).

**Methods:**

This retrospective monoinstitutional cohort study included 49 consecutive patients in total with a newly diagnosed rectal cancer. All the patients underwent baseline abdominal MRI and CT scan of the chest and abdomen to exclude distant metastasis before C-RT. Texture parameters were extracted from MRI performed before C-RT (T1, DWI, and ADC sequences) using LifeX Software, a dedicated software for extracting texture parameters from radiological imaging. We divided the cohort in a training set of 34 patients and a validation set of 15 patients, and we tested the data sets for homogeneity, considering the clinical variables. Then we performed univariate and multivariate analysis, and a ROC curve was also generated.

**Results:**

Thirteen patients (26.5%) showed an ePD, three of whom with lung metastases and ten with liver relapse. The model was validated based on the prediction accuracy calculated in a previously unseen set of 15 patients. The prediction accuracy of the generated model was 82% (AUC = 0.853) in the training and 80% (AUC = 0.833) in the validation cohort. The only significant features at multivariate analysis was DWI GLCM Correlation (OR: 0.239, *p* < 0.001).

**Conclusion:**

Our results suggest that TA could be useful to identify patients that may develop early progression.

## 1. Background

Neoadjuvant radiation therapy (RT) in combination with chemotherapy (fluoropyrimidines alone or in association with oxaliplatin) is commonly used in patients with locally advanced rectal cancer (LARC) before radical surgery [1–3]. Chemoradiotherapy (C-RT) decreases the rate of local relapse and prolongs the progression-free survival (PFS) of these patients and seems to have an effect also on the overall survival (OS) [4–8].

The results of previous studies suggest the reliability of local staging with pelvic magnetic resonance imaging (MRI) in defining circumferential tumor margin involvement, extramural vascular invasion, and locoregional nodes involvement [9, 10]. These parameters are, in turn, strictly correlated with a high risk of relapse. Additionally, the use of diffusion-weighted imaging (DWI), which defines tumor tissue cellularity, has shown promising results in predicting treatment response to C-RT [11, 12]. Recently, texture analysis (TA) has been developed to improve the evaluation of tumor sites by quantifying heterogeneity and to identify the parameters to add more information for local staging [10]. This analysis, refers to multiple mathematical models, aimed at providing a reliable measurement of heterogeneity within a selected image (texture features). It is performed by measuring the means of a computer quantification of both gray-level intensity, position of pixels, and its use is being investigated in several fields [13] as well as monitoring and research of biomarkers in cancer patients [14–16]. On these bases, we have designed the present study to evaluate the potential use of MRI TA to predict the outcome and relapse of LARC patients undergone C-RT.

## 2. Methods

### 2.1. Patients

We performed a retrospective analysis of rectal cancer patients consecutively treated with C-RT at our Radiation Oncology Unit between January 2010 and December 2015. Inclusion criteria were achievable endoscopic/bioptic diagnosis and a complete clinical-radiological pretreatment staging (including chest-abdomen CT and abdomen MRI), in order to exclude systemic metastases at referral, standard long-course neoadjuvant C-RT [17, 18], MR imaging examination after the end of C-RT, surgical treatment by TME, and the evaluation of tumor regression grade in the postsurgical report. Examinations without DWI acquisition and patients without post-C-RT MR examination were excluded. All the patients gave written consent to anonymous use of their examinations for research scope, and a notification of the study was submitted and approved by the local ethical committee of our institution, as established by the national laws. All the clinical management of the patients was performed in accordance with relevant international guidelines [19] as follows.

### 2.2. C-RT Therapy Protocols

Radiation therapy volumes included the rectum, mesorectum, presacral nodes, and internal iliac nodes. A radiation dose of 45 Gy was delivered with conformational radiation technique (3D-CRT) in 5 weekly sessions of 1.8 Gy/daily for 5 weeks. Intensity-modulated RT (IMRT) technique was used in selected cases requiring a more refined technique.

A further boost dose of 5.4 Gy in three sessions (9 Gy in five sessions for T4 patients) was delivered the following week and coned down to tumor with a 2 cm margin and adjacent mesorectal region, with 15 MeV photons and a 3D-CRT technique. Capecitabine (825 mg/m^2^, twice daily for five days/week) was delivered daily throughout the radiotherapy course for five weeks [17, 18].

### 2.3. Magnetic Resonance Imaging

A pelvic MRI examination (1.5-T system, Signa Excite HD, GE Healthcare, Milwaukee, WI, USA) with an eight-channel phased-array coil was obtained in all patients at baseline and 30 ± 15 days after the end of C-RT. The imaging protocol consisted of high resolution fast spin echo (FSE) T2-weighted sequences in almost 3 planes oriented perpendicularly and parallel to the axial extension of the lesion in the rectal lumen [20]. DWI based on echo-planar spin-echo (SE-EPI) sequence, with 90° and 180° radiofrequency pulse series application, was also acquired.

A fat-saturated pulse was always applied to avoid chemical shift artifacts. This sequence was acquired in the same axial oblique plane of the T2-weighted images by application of a b-factor 0 and 800 s/mm^2^ (DW images) on three orthogonal directions. Finally, ADC maps were extrapolated from DWI data set by a commercial software (Functool 4.4, GE Healthcare, USA).

### 2.4. Surgical Treatment and Histopathological Assessment

All included patients underwent TME after 50 ± 18 days after the end of C-RT. An expert pathologist examined surgical specimens, according to the AJCC Cancer Staging Manual (8th edition, 2017), and the tumor regression grade (TRG) was established according to Dworak score: grade 0, no regression; grade 1, dominant tumor mass with obvious fibrosis; grade 2, dominantly fibrotic changes with few tumor cells or groups; grade 3, very few tumor cells; grade 4, no tumor cells [21].

### 2.5. Follow-Up

After the completion of the multimodal treatment, patients entered a scheduled follow-up program, according to the institutional guidelines, with a CT scan and/or MRI repeated, in order to assess the recurrence of the pathology, at 9-12 weeks after the surgery, and then every 6 months for the first two years or in any case showing clinical signs suggesting progressive disease (PD). General examinations with recording of toxicity, blood cell counts and chemistry, and serum CEA levels were evaluated every three months for the first two years.

### 2.6. Contouring

**Figure 1:**
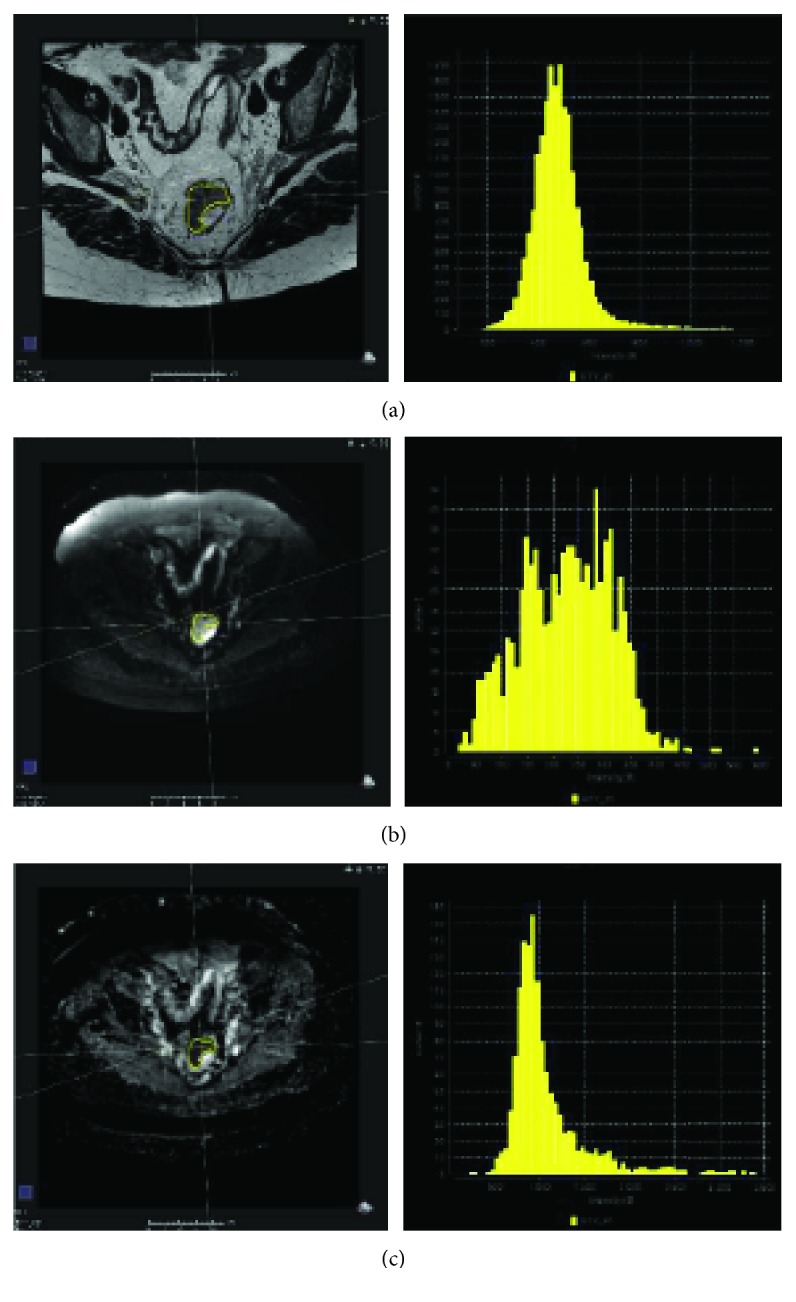
The gross tumor volume (GTV) was evaluated at baseline by MRI and contoured on T2 (a), DWI (b), and ADC (c) sequences.

The gross tumor volume (GTV) was contoured on *pre-C-RT MRI (baseline)* by a radiation oncologist (VN) and confirmed by one expert radiologists (SFC) on T2, DWI, and ADC sequences (Figure 1).

The contouring was performed on each slide where the GTV was visible, in order to obtain a 3D region of interest (ROI) that was used to extract TA parameters.

The operators had all the access to clinical information, including imaging pre-C-RT. The impact of variations on contouring was analyzed performing two delineation on each patients, and the TA parameters were tested for reliability with intraclass coefficient correlation (ICC) method. All the analysis for this work have been accomplished with LifeX Software©. TA parameters included the features of gray-level cooccurrence matrix (GLCM), compacity, sphericity, and indices from the gray-level histogram.

## 3. Preselection of Variables, Endpoints, and Statistical Analysis

The reliability analysis performed with ICC showed that the TA parameters significantly reproducible (ICC > 0.70, single measure) were, respectively, 4 out of 13 for the T2-MRI (30%), 10 out of 13 (77%) for the DWI-MRI, and 11 out of 13 (84%) for the ADC-MRI (Table 1). The cohort was randomly split in a training set of 34 patients and a validation set of 15 patients. In order to perform a statistical correlation among the reliable TA parameters and outcome, we chose as a statistical endpoint the occurrence of an early progression of disease (ePD) defined as the percentage of patients who relapsed or showed distant metastasis within three months from the radical surgery (confirmed at consecutive follow-up examinations with CT scan, repeated at 1 month). These TA parameters and multiple known prognostic factors (grading, stage of disease, and TRG) were then correlated with ePD by performing a univariate analysis (univariate logistic regression, with Bonferroni correction for the number of variables). We analyzed the correlation between the significant TA parameters, and if a correlation larger than 0.80 was observed (Pearson correlation), then the variable with the lowest univariable correlation with the ePD was omitted to avoid the risk of overfitting the model and of multicollinearity [22] in the multivariate analysis (binary logistic regression). Logistic regression analysis was optimized by using the training data set, and the outcome of the testing data was then predicted within the optimized model also in the validation cohort. The prediction results were further interpreted using the receiver operating characteristic (ROC) curve. All the statistical analysis was conducted with SPSS software 23.0.

## 4. Results

Finally, we included 49 patients in our study, 34 (69%) males and 15 (31%) females with a median age of 67 years (mean ± SD = 66.9 ± 10 years, range 38-85 years).

A total of 12 patients were excluded from the analysis since they did not respond to the inclusion criteria.

All patients were submitted to the follow-up (FU) program in our institute; the median time of FU was 38 months (±14 months). 11 pts (22%) had complete response at pathological examination (TRG 4), whereas 26 (53%) showed to be good responders (TRG 3-4).

The results of clinical staging before and after C-RT and of the resected specimen are provided in Table 1.

According to the type of surgery, 20 patients underwent low anterior resection (LAR) and 29 patients underwent abdominoperineal resection surgery (APR). The median number of nodes removed was 12 (mean 13.7 ± 5.8, range 6-30), and the ratio of positive lymphonodes/total nodes was between 1/20 and 11/19 (median value 2/18).

The median circumferential resection margin (CRM) was 6 millimeters (mean 7.4 ± 6 mm, range 0.1-20 mm).

**Figure 2:**
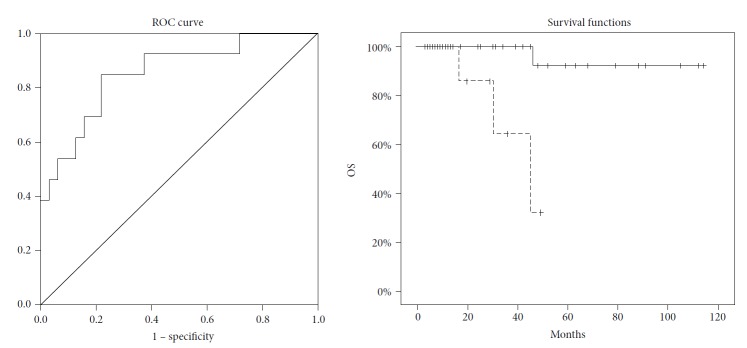
ROC curve for the prediction of early progression of disease (ePD) (left) and Kaplan-Meier OS curve for the two subgroups (continuous line: no ePD, 108 ± 5 months, 95% CI 98-118 line, ePD: 38 ± 4 months, 95% CI 28-48 months, *p* < 0.001).

There was a 26% rate of ePD (thirteen patients) with occurrence of metastases at the lung (three patients) and liver (ten patients). There was a substantial difference in OS when patients with ePD were compared with the other group of patients (no ePD: 108 ± 5 months, 95% CI 98-118 months vs. ePD: 38 ± 4 months, 95% CI 28-48 months, *p* < 0.001) (Figure 2).

**Figure 3:**
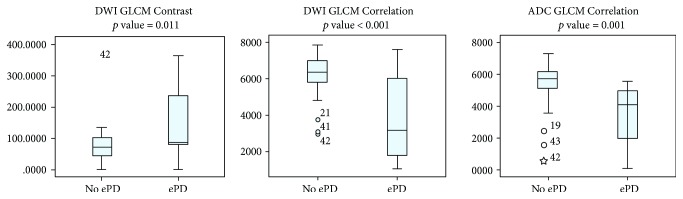
BoxPlot of the TA parameters in the two subgroups of patients who develop (ePD) or not develop (no ePD) early distant progression.

Our univariate statistical analysis (one-way ANOVA, with Bonferroni correction) revealed a significant correlation of ePD with the following TA parameters: DWI GLCM Contrast (OR: 2.40, *p* = 0.009), DWI GLCM Correlation (OR: 0.239, *p* < 0.001), and ADC GLCM Correlation (OR: 0.318, *p* = 0.001) (Figure 3). In particular, GLCM parameter Contrast, on DWI, was significantly higher in patients developing ePD, and conversely, GLCM Correlation, on DWI and ADC, was found significantly lower in patients developing ePD.

No one of the clinical parameters was correlated with ePD, at both univariate analysis and multivariate analysis.

Multivariate logistic regression analysis also showed a strong correlation among DWI GLCM Correlation (*p* = 0.001, OR: 0.002, 95% CI: 0.001-0.175, overall AUC: 0.853, 95% CI 0.728-0.979), with an overall *R*^2^ of 0.350 (Figure 2). The characteristics of the ROC curves included 2 log-likelihood (49.715), Nagelkerke *R*^2^ (0.391), AUC (0.853), and Hosmer–Lemeshow (Chi-square: 6.496, *p* value = 0.483). By the analysis of the ROC curve, we chose 0.400 as the cut-off value of the parameter DWI GLCM. Correlation has a sensibility of 93.8%, a specificity of 53.2%, and a positive predictive value of 82.2% for the prediction of early progression. The binary logistic regression analysis was repeated with the validation set, and the overall AUC was equal to 0.833, 95% CI 0.619-1.000 (Table 2).

## 5. Discussion

The results of our study suggest that a noninvasive and low-cost mathematical analysis of MRI, such as TA, could be used to identify the patients with LARC with a higher risk of developing distant metastases and, therefore, may need additional systemic treatments over the classical neoadjuvant C-RT. In fact, the clinical development of new generation agents, such as anticancer mAbs, new target-driven drugs, and immunotherapy in the treatment of several malignancies has ignited the research of new predictive biomarkers on imaging, histological sample, liquid biopsies, and other serum personalized markers [23].

At this regard, many authors have already investigated the possible correlation of imaging techniques, especially MRI (volumetric analysis and TA) with patients' outcome; however, all of them mainly used as an effective statistical endpoints the achievement of a complete pathological response (pCR) [10–12, 14–16, 24–26].

Our results have shown that the GLCM parameter Contrast was significantly higher in patients developing ePD. Conversely, GLCM Correlation, on DWI and ADC, was found significantly lower in patients developing ePD.

The GLCM parameter, in line with the results by Esgiar and coll. [27], is considered as an efficient TA method. It is based on a second-order statistics able to characterize the properties of two or more pixel values in specific sites. An early study by Esgiar et al. already showed that entropy texture features extracted from GLCM was capable of differentiating between normal and neoplastic tissue [27]. Additionally, by performing color channel histograms, GLCM, and structural features, Kalkan et al. achieved an accuracy of 75.15% in the classification of four types of colon tissues: normal, cancerous, adenomatous, and inflammatory [28]. In our study, a higher GLCM Contrast and a lower GLCM Correlation are also in line with the recent results by Chaddad et al., who report the use of TA extracted from multispectral optical microscopy images for the classification of three types of pathological tissues: benign hyperplasia, intraepithelial neoplasia, and carcinoma [29]. The latter authors in fact report that a higher GLCM Contrast and a lower GLCM Correlation are correlated to higher aggressiveness of the tumor. By continuing on the same track, Nie et al. report that LARC patients who did not achieve a complete pathological response upon neoadjuvant C-RT present a higher Contrast and a lower homogeneity [16]. These findings suggest that these parameters can be, therefore, related to a lower sensitivity to C-RT of these patients who therefore present a higher risk of early development of distant metastases. To the best of our knowledge, this is the first attempt to predict an early progression of disease using the clinical, anatomical, and multiparametric MRI with volumetric TA.

Actually, the knowledge of the pathophysiological mechanisms that are correlated with the radiomics analysis is unknown. Some authors have correlated the TA parameters to the areas of the tumor with specific metabolic hallmark (e.g., hypoxia). Further studies are needed to test these hypotheses at a cellular level (e.g., vascularization, receptor expression, and angiogenesis).

Our results show that the volumetric TA could provide additional information in assessing the risk of developing distant treatment failure and can be used to identify the patients at risk, providing also a cut-off value of TA parameter, although it should be validated with an external data set. At the same time, the patients with ePD, in respect to the patients without ePD, show a significant lower survival.

## 6. Limitations of the Study

Our results may be worthy of critical consideration for possible methodological and technical refinements. In particular, our study has the limitations of a monoinstitutional retrospective study, and the correlations between textural parameters and clinical outcome need further investigation, in order to better understand the pathological basis of the TA parameters. Further, we need to investigate the real reproducibility and the reliability of this kind of analysis in other departments and hospitals, with different parameters of MRI acquisitions. We finally recognize as a limitation the low number of patients enrolled.

## 7. Conclusions

Early progression of disease represent an important cause of progression for patients with rectal cancer undergoing neoadjuvant C-RT as the locoregional relapse is very rare. Our results appear to be promising since the TA seems to improve the knowledge of the predictive factors of this kind of progression of disease and may lead to different approaches to this subset of patients, such as intensive neoadjuvant chemotherapy.

Further studies on a large population with external validation are needed to best estimate the present preliminary data.

Texture analysis could represent in the next future a promising imaging biomarker of cancer aggressiveness, although these results need to be further validated.

Our results could help in providing additional information in assessing the risk of developing distant treatment failure and could be used in the future to identify the patients at risk.

## Figures and Tables

**Table 1:** Characteristics of patients.

Characteristic	Numbers (%)
Sex	
Males	34 (69%)
Females	15 (31%)
Age	
<70 years	29 (61%)
>70 years	20 (39%)
Clinical staging before C-RT	
(T)	
cT2	10 (20.4%)
cT3	34 (69.4%)
cT4	5 (10.2%)
(N)	
cN0	5 (10.2%)
cN1	33 (67.3%)
cN2	11 (22.4%)
UICC	
Stage II	5 (10.2%)
Stage III	44 (89.8%)
(T)	
cT0	3 (6.2%)
cT1	1 (2%)
cT2	25 (51%)
cT3	19 (38.8%)
cT4	1 (2%)
(N)	
cN0	40 (81.6%)
cN1	7 (14.3%)
cN2	2 (4.1%)
UICC	
Stage 0	3 (6.1%)
Stage I	20 (40.8%)
Stage II	17 (34.7%)
Stage III	9 (18.4%)
Surgery	
LAR	20 (40.8%)
APR	29 (49.2%)
(yT)	
yT0	7 (14.3%)
yT1	6 (12.3%)
yT2	15 (30.7%)
yT3	20 (40.7%)
yT4	1 (2%)
(yN)	
yN0	35 (71.4%)
yN1	10 (20.3%)
yN2	4 (8.3%)
UICC	
Stage 0	11 (22.4%)
Stage I	12 (24.5%)
Stage II	17 (34.7%)
Stage III	9 (18.4%)
Grading biopsy before C-RT	
G1	3 (6%)
G2	37 (75%)
G3	9 (18%)
TRG	
4	11 (22%)
3	15 (31%)
2	20 (41%)
1	3 (6%)

**Table 2:** Univariate and multivariate analysis.

Endpoint	Parameter	*p* value	Β	OR (95% CI)
Univariate analysis				
ePD	DWI GLCM Contrast	0.040	0.875	2.40 (1.04-5.54)
	DWI GLCM Correlation	<0.001	-1.431	0.239 (0.09-0.61)
	ADC GLCM Correlation	0.009	-1.144	0.318 (0.13-0.74)
Multivariate analysis				
ePD	DWI GLCM Correlation	0.001	-1.371	0.254 (0.10-0.59)

## Data Availability

The data used to support the findings of this study are included within the article.
